# Agronomic and Quality Traits of 30 Eggplant Germplasm Resources from China

**DOI:** 10.3390/plants14121838

**Published:** 2025-06-15

**Authors:** Jian Lyu, Li Jin, Xianglan Ma, Yansu Li, Mintao Sun, Ning Jin, Shuya Wang, Linli Hu, Jihua Yu

**Affiliations:** 1 College of Horticulture, Gansu Agricultural University, Lanzhou 730070, China; lvjiangs@126.com (J.L.); mxl202208@163.com (X.M.); jinn0513@163.com (N.J.); hull@gsau.edu.cn (L.H.); 2 State Key Laboratory of Aridland Crop Science, Gansu Agricultural University, Lanzhou 730070, China; jinli0124@163.com (L.J.); wsyhn95@163.com (S.W.); 3 Vegetable and Flower Research Institute of Chinese Academy of Agricultural Sciences, Beijing 100081, China; liyansu@caas.cn (Y.L.); sunmintao@caas.cn (M.S.)

**Keywords:** eggplant, morphological characters, nutritional quality, Solanaceae, varietal diversity

## Abstract

(1) Background: Eggplant is a widely grown, high-value vegetable crop whose commercial demand has increased in recent years owing to its unique nutritional features. Variations in its agronomic and nutritional traits are of great importance in the selection of eggplant varieties. (2) Methods: In this study, 30 different eggplant varieties were evaluated concerning the morphological characteristics and nutritional value of their fruits. (3) Results: Among the eight morphological characteristics evaluated, the coefficient of variation was highest for fruit calyx thorns, pericarp brightness, and fruit shape index. The diversity index (H’) for pulp color was the largest, followed by pericarp brightness, but was the smallest for fruit weight. Principal component analysis showed that the morphological characteristics contributed 73.20% for the observed diversity among the 30 eggplant varieties, whereas eggplant fruit quality traits had a minor effect. Of note, significant differences in the soluble protein, vitamin C, nitrate, soluble sugar, organic acid, and mineral contents was observed within the samples, with organic acids, vitamin C, and hardness contributing more to the total variation observed. Multiple sets of correlations among the indices were found, with significant positive correlations between transverse diameter and hardness, fruit weight and fruit shape index, as well as between malic acid, fructose, and sucrose; (4) Conclusions: Altogether, these findings may help create breeding strategies to promote the selection of superior genotypes and help guide future germplasm collection.

## 1. Introduction

Eggplant (*Solanum melongena* L.), which belongs to the Solanaceae family, is an important vegetable crop commonly cultivated in subtropical and tropical regions that is economically important in Asia, Africa, and Europe. In terms of yield and harvested area, eggplant ranks third among cultivated solanaceous crops, after tomato and potato. Indeed, eggplants are considered to be a valuable vegetable crop because it contain high concentrations of bioactive compounds that provide nutritional benefits [1,2]. Namely, they were reported to hold therapeutic potential for anemia, atherosclerosis, and steatosis, with its antioxidant properties also helping to reduce the risk of various cancers, prevent cardiovascular diseases, and protect against acute respiratory infections [3,4,5,6]. Eggplants are rich in polyphenols, which can help protect cell membranes and enhance memory function in the brain [7], and also in anthocyanins, which are important for the treatment of diabetes, neurological problems, cardiovascular diseases, and cancer. For example, the pulp of purple eggplant contains high levels of nasunin that can help prevent lipid peroxidation and reactive oxygen species accumulation in cells owing to its antioxidative properieties [8]; moreover, it can also act as an iron chelator. Moreover, eggplants are rich in carbohydrates, proteins, vitamins, and minerals, such as zinc, iron, magnesium, manganese, potassium, and copper, which are important for bone health. Indeed, eggplants can also be used to treat premenstrual syndrome, amenorrhea, and prenatal anemia owing to its iron content [9,10]. A previous study showed that dry eggplant can be used to treat gastric distension and hemorrhoids, whereas fresh eggplant can help strengthen the bones, control diabetes, and prevent paralysis [11]. In addition, eggplant fiber can aid digestion by removing toxins and harmful substances from the stomach [12]. Despite the vast nutritional and medicinal qualities of eggplants, many of their bioactive compounds have not yet been effectively characterized. Therefore, it is critical for eggplant breeders to balance the yield, taste, appearance, and bioactive compound content to obtain optimal results [13].

Owing to its unique economic and nutritional value, the eggplant demand has increased globally in recent years, but its breeding remains limited [14]. Eggplants are planted worldwide in the wild form [15], with thousands of varieties being locally cultivated and commercialized [16]. Overall, domestication, mutation, natural hybridization, human selection, and crossbreeding have allowed eggplants to acquire a wide range of genetic diversity, improving their sustainable production. Moreover, it helps them adapt to climate change challenges [17]. In addition to cultivated varieties, wild relatives of eggplants are important sources of useful genes and underutilized traits, such as disease resistance, drought tolerance, and high bioactive compound content [18]. Morphological screening is the first step for identifying genetic variation in eggplants, with morphological characteristics being the main criteria for eggplant classification [19]. Characterization using traditional morphological descriptors can also be useful for describing and establishing relationships among local eggplant genetic resources [20]. As the nutritional and agromorphological characteristics of eggplants vary considerably, selection of the available materials with the desired traits and identification of their morphological descriptors are key steps for the breeding process [21]. However, previous studies on eggplant breeding have only focused on the use of high-yield varieties and hybrids to meet the global consumption needs [22].

Based on the current developments in the eggplant industry and consumer consumption habits, this study collected 30 eggplant varieties from 10 main eggplant breeding units nationwide. These varieties cover all eggplant types in the national market. By evaluating their morphological and nutritional quality, the study aims to offer strong evidence for selecting high quality eggplant varieties.

## 2. Results

### 2.1. Appearance and Morphology of Chinese Eggplant Fruits

The fruit calyx thorn, peel brightness, and fruit shape index differed markedly among the 30 eggplant varieties fruit evaluated, whereas the fruit calyx thorn, pulp color, peel color, transverse diameter, and fruit shape index were similar among the samples (Table 1). Accordingly, among all the measured appearance traits, the 30 eggplant varieties’ fruit shape index showed the highest coefficient of variation, followed by the 30 eggplant varieties’ fruit calyx thorn and peel brightness, whereas the 30 eggplant varieties’ fruit longitudinal diameter showed the smallest coefficient of variation. Pulp color showed the largest index of morphological diversity (H’) among 30 eggplant varieties tested, followed by peel brightness, and fruit weight showed smallest H’ index.

### 2.2. Saccharides Content in Chinese Eggplant Fruits

**Figure 1:**
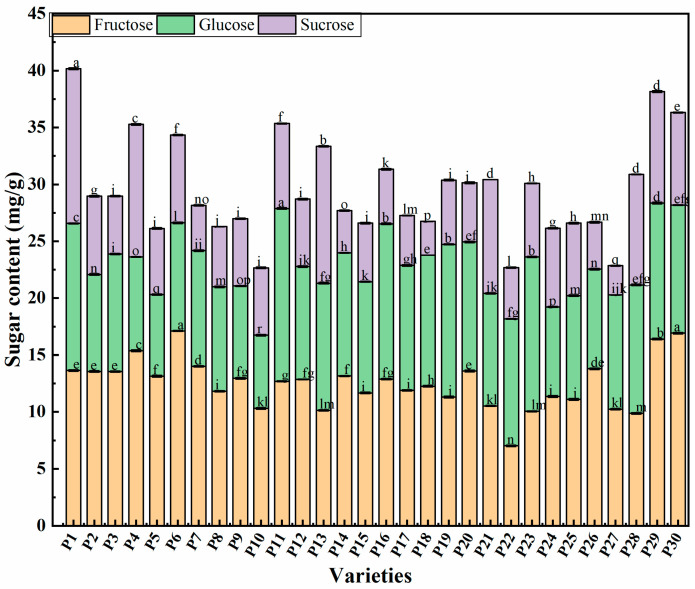
Saccharide content in 30 eggplant varieties from China. P1–P30 represent the different eggplant varieties evaluated. Data are expressed as mean values ± standard errors of three replicates (*n* = 3). Different lowercase letters represent significant differences (*p* < 0.05).

Fructose, glucose, and sucrose were the predominant sugar components found in the 30 eggplant fruits evaluated (Figure 1). Among them, P6 had the highest fructose content, followed by P30, differing significantly from the remaining samples (Table S2). The fructose content of P22 (7.02 mg·g^−1^) was significantly lower than that of the other varieties. The glucose content of the P11 variety was significantly higher than that of the other varieties, followed by P16 (13.65 mg·g^−1^), whereas P5 had significantly lower glucose levels than the other varieties. Most of the 30 eggplant varieties had a lower sucrose content than fructose and glucose.

### 2.3. Organic Acid Components Content in Chinese Eggplant Fruits

**Figure 2:**
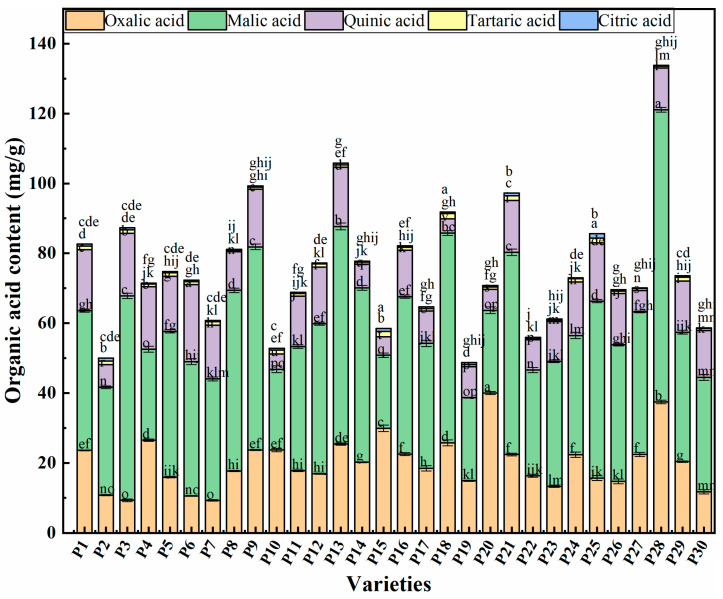
Content of organic acids in Chinese eggplant varieties. P1–P30 represent the 30 different eggplant fruit varieties evaluated. Data are represented as mean ± standard error of three measurements (*n* = 3). Different lowercase letters represent significant differences (*p* < 0.05).

The eggplant fruit varieties evaluated showed significant differences in the contents of organic acid components (Figure 2). Among them, P20 had significantly higher oxalic acid content than the other varieties, which was 3.31-times higher than that of the lowest value detected in P7 (Table S3). Malic acid was the most common organic acid detected in all 30 eggplant varieties, with P28 showing the highest levels among all samples, which was 3.00-times higher than that of the sample with the lowest malic acid content (P15). Moreover, quinic acid was detected in high levels in P6, which was 4.43-times higher than the lowest content detected in P18. The highest content of tartaric acid was found in P15, which was similar to the amount detected in P18, being 2.74-and 2.64-times higher than the lowest content in P22. Lastly, P25 was found to have significantly higher content of citric acid than that of the other varieties, being 8.36 times higher than the lowest content of P27.

### 2.4. Other Quality Indicators in Chinese Eggplant Fruits

**Figure 3:**
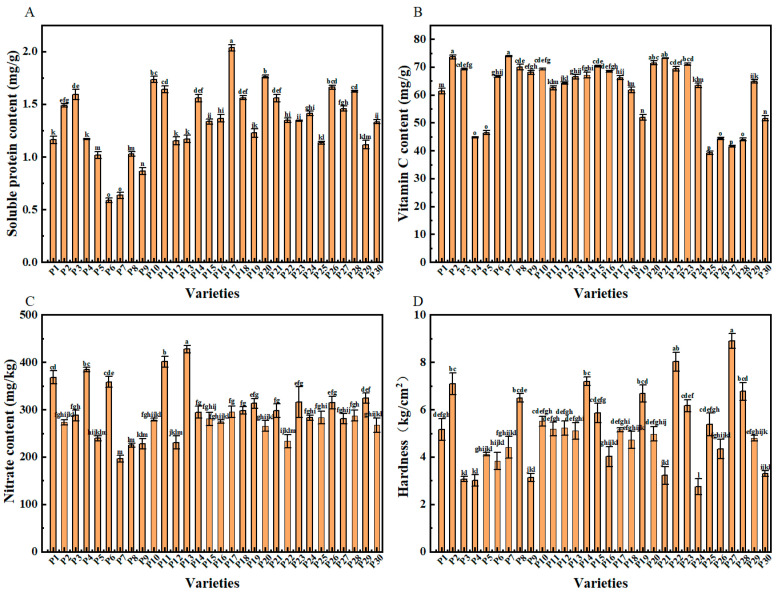
Contents of (**A**) soluble proteins, (**B**) vitamin C, (**C**) nitrates, and (**D**) fruit hardness of different Chinese eggplant fruits. P1–P30 represent the 30 eggplant fruit varieties evaluated. Data are represented as average values ± standard errors of three replicates (*n* = 3). Different lowercase letters represent significant differences (*p* < 0.05).

The soluble protein content differed significantly among the 30 eggplant fruit varieties (Figure 3A). Among them, P17 had the highest soluble protein content, with 2.03 mg·g^−1^ among all evaluated samples, whereas P6 and P7 had 71.11% and 68.84% lower soluble protein content than P17, respectively. In turn, P7 (73.86 mg·g^−1^) had the highest amount of vitamin C, followed by P2 (73.63 mg·g^−1^), values that differed significantly from those detected in all the other samples (Figure 3B). The variety with the lowest vitamin C content was P25, which was similar to that of P27. Overall, the vitamin C content of the P7 was 98.69% and 77.96% higher than that of the P25 and P27, respectively, whereas that of P2 was 98.04% and 77.42% higher than that of the P25 and P27, respectively. The variety with the lowest nitrate content was P17 with 195.86 mg·kg^−1^, followed by P8 (Figure 3C). Except for varieties P11 and P13, the nitrate content of all eggplant fruits evaluated was below 385 mg·kg^−1^. Significant differences were also observed concerning the hardness of the fruits (Figure 3D), with P27 being the hardest sample among all tested, followed by P22, whereas P24 was the softest of all fruits, being 69.11% softer than P27.

### 2.5. Mineral Element Content in Chinese Eggplant Fruits

**Figure 4:**
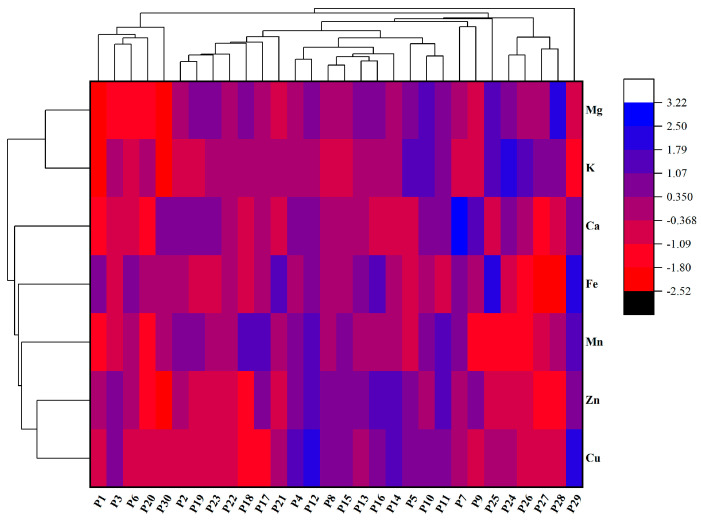
Cluster heatmap analysis of the mineral contents of the Chinese eggplant fruits. P1–P30 represent the 30 different eggplant varieties evaluated. The data are expressed as average values (*n* = 3).

Among the 30 eggplant fruit varieties evaluated, P29 and P18 had the highest and lowest Cu content, respectively, with a 2.27-fold difference (Figure 4). In turn, Fe content was highest in P25, which differed 1.34-fold than that of P28. P18 had the highest Mn content among all samples, which was 1.11 times higher than that of P24 variety. P16 variety had the highest Zn element content, which was 33.07% higher than that of the lowest content of P30. P24 had the highest K element content, which was 33.07% higher than that of P30. P7 had the highest Ca content among all samples, which differed by 2.08-fold from the sample with the lowest content (P1). Mg content was found to be highest in P28, being 1.05-times higher than that of the sample with the lowest value (P1).

### 2.6. Comparative Assessment of the Appearance and Nutritional Traits of Chinese Eggplant Fruits

**Figure 5:**
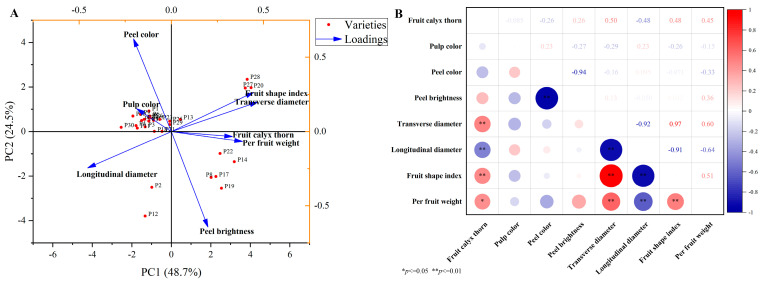
(**A**) Principal component analysis and (**B**) correlation analysis of the appearance traits of the Chinese eggplant varieties. P1–P30 represent the 30 different eggplant varieties evaluated. The data are expressed as average values (*n* = 3). The * and ** represent significant correlations at the *p* < 0.05 and *p* < 0.01 levels, respectively (two-tailed).

PCA was performed on all samples based on the above reported characteristics of the 30 eggplant varieties (Figure 5A). Overall, PC1 and PC2 were found to explain 48.70% and 24.50% of the variability, respectively, successfully separating all tested eggplant varieties into four groups regarding their appearance traits. Specifically, P20, P27, and P28; P2 and P12; P8, P14, P17, P19, and P22 clustered into three separate groups, whereas the remaining varieties were grouped together. Correlation analysis of the appearance characteristics of all eggplants tested revealed highly significant positive correlations (*p* < 0.01) between fruit calyx thorn, fruit shape index, and transverse diameter (Figure 5B). Transverse diameter showed a highly significant positive correlation (*p* < 0.01) with the fruit weight and shape index. In contrast, peel color showed a highly significant negative correlation with peel brightness, and longitudinal diameter also showed a highly significant negative correlation with fruit shape index and per fruit weight (*p* < 0.01).

**Figure 6:**
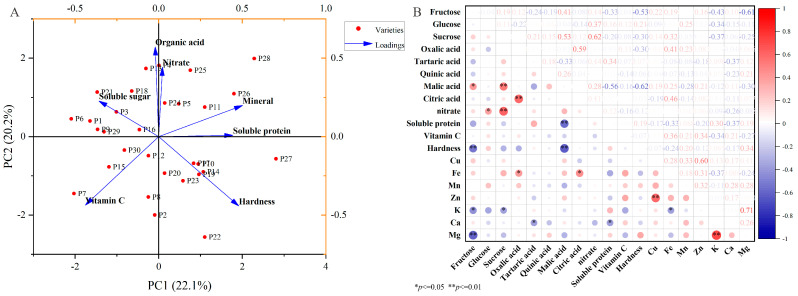
(**A**) Principal component analysis and (**B**) correlation analysis of the nutritional quality of the Chinese eggplant varieties. P1–P30 represent the 30 different eggplant varieties evaluated. The data are expressed as average values (*n* = 3). The * and ** represent significant correlations at the *p* < 0.05 and *p* < 0.01 levels, respectively (two-tailed).

Further PCA concerning the nutritional quality indicators of all 30 eggplant varieties revealed that PC1 and PC2 explained only 22.10% and 20.20% of the variability, respectively (Figure 6A). Indeed, components 1 and 2 failed to effectively separate the eggplant fruit varieties into groups. Nevertheless, correlation analysis revealed a positive correlation between malic acid and fructose (*p* ≤ 0.05) and a highly significant positive correlation between malic acid and sucrose (*p* ≤ 0.01) (Figure 6B). A highly significant positive correlation was also observed between the oxalic and citric acids levels (*p* < 0.01), and between Cu and Zn, and K and Mg (*p* < 0.01). In contrast, fructose and Mg, and malic and soluble protein contents were negatively correlated (*p* < 0.01).

## 3. Discussion

Recent studies have shown that eggplants have undergone more than one domestication process, which involved an increase in fruit size and changes in fruit color and shape [17]. For example, the eggplant fruit’s shape has changed from elongated to oval. Notably, changes in the intensity of the color purple in the fruits is known to be related with genotype, probably because of the presence of different types of chlorophylls and anthocyanins in the fruits [23,24]. Fruit shape, peel color, fruiting habit, and growth habit are all traits that determine the quality of eggplants and are considered for further improvement of eggplants cultivation. Fruit shape and peel color are standard appearance characteristics. In the present study, 10 round-fruited and 20 long-fruited eggplant varieties were selected as the study materials, and both fruit types contained two peel colors: purple and green. Descriptive statistical analysis of the 30 eggplant varieties tested showed that the fruit calyx thorn, peel brightness, and fruit shape index, which are traits important for documentation, conservation, and crop improvement of this species, varied over a wide range among all samples (Table 1). The prickles and pubescence of plant parts are primarily used in breeding programs to reduce the incidence of pests and associated diseases. The presence of spines in the calyx is associated with good sensory qualities [25]. In contrast, the pubescence of some plants causes a strong sensation in the skin and adversely affects the respiratory system of people who come into contact with the plant. We found that among 30 eggplant varieties, round eggplants generally have a higher number of fruit calyx thorn than long eggplants. Therefore, these important traits must be considered during parental selection. In addition, the Shannon-Weaver diversity index (H’) is one of the most commonly used methods for assessing the diversity of germplasm resources and it is often used to define plant phenotypic characteristics and metabolite diversity [26,27]. Herein, the maximum H’ index was observed for the pulp color, followed by peel brightness, whereas the lowest H’ value was that of the fruit weight. Overall, these traits contribute to the genetic diversity of eggplants and thus genotypes related to these traits should be identified and documented. Hardness is also a quantitative indicator of texture in the sensory requirements of eggplant fruits, implying that its skin is thick, which in turn may contribute for a poorer taste poor and, consequently, impact on its commercial value. Therefore, significant differences in fruit hardness among the 30 eggplant varieties herein described provide new insights for breeders.

The modification of agronomic traits in eggplants is an important aspect of breeding. However, in the last few years, in response to consumer demand for fruits and vegetables of high quality, researchers have studied sensory and nutritional characteristics, bioactive and anti-nutritional compounds, postharvest and processing-related traits of eggplant fruits from a genetic/genomic perspective [28,29,30]. Among Solanaceae species, most studies have focused on the biochemical characterization and nutritional value of tomato [31], whereas the genetic basis of tuber quality and alkaloid content has been investigated in potato [32]. In contrast, studies on eggplants have focused on their polyphenols, anthocyanins, chlorogenic acids [33], and alkaloids contents [34]. To date, information is lacking regarding health-related metabolites of eggplants, namely regarding their sugar and organic acids content, despite their key role in breeding programs. In the present study, fructose and glucose contents were found to be generally higher than that of sucrose, thereby being major contributors for eggplant sweetness. The fructose and glucose contents varied among the 30 samples of eggplant fruits tested. Overall, purple round eggplants generally have higher fructose levels than purple long eggplants, while purple long eggplants usually contain more glucose than purple round ones. Meanwhile, green long eggplants have higher levels of fructose, glucose, and sucrose compared to green round eggplants. Our evaluation of the organic acid components of the 30 eggplant varieties also revealed that malic acid was the most abundant organic acid component in eggplant fruits. Malic acid is known for its potential to aid muscle recovery (used in the treatment of fibromyalgia), ameliorate fatigue, and increase energy. In addition, it is often used by the food industry as an acidifier, flavoring agent, and stabilizer, and by the pharmaceutical industry for cleansing and regenerating wounds and burns [35]. The eggplant varieties with the highest organic acid content had 2–8-times higher concentrations of these compounds than the varieties with the lowest organic acid content, an observation that agreed with of Silva et al. [36]. Commonly, differences in the organic acid content of eggplants are mainly due to the variety, cultivation conditions, and geographical region.

Season, environment, and genotype strongly influence the carbohydrates, starch, vitamin C, and phenolics contents in the eggplant fruit. Similar results to our study were reported in studies of dry matter, protein, total phenolics, mineral content, and lycopene [33]. There are significant differences in the fruit’s soluble protein and vitamin C content among the 30 eggplant varieties. Varieties P7 and P17 have the highest levels of these nutrients, with increases of 244.07% and 46.98% respectively over the lowest–content varieties. This shows that the soluble protein and vitamin C content in eggplants is genotype–dependent. This is in line with the findings of Martínez-Ispizua et al. [37], who studied local eggplant varieties. Vitamin C is a key antioxidant. The relatively low vitamin C content in eggplant fruits might limit the plant’s overall antioxidant capacity [38]. In contrast, nitrate reacts rapidly in the body to form metabolites, such as nitrite, nitric oxide, and N-nitroso, that may pose a direct threat to human health [39]. Of note, we found that the nitrate content differed markedly among the 30 eggplant varieties evaluated.

Mineral elements play important roles in maintaining healthy human physiological functions, as they help stabilize proteins and are cofactors of several enzymes. Certain mineral elements regulate key biological processes by binding to receptor sites in cell membranes or altering the shape of receptors to prevent specific molecules from entering the cell [40]; thus, their deficiency can lead to diseases [41]. The eggplant fruits varieties evaluated were found to contain Cu, Fe, Mn, Zn, K, Ca, and Mg. Except for K, the varieties with the highest mineral contents had 1–2-times higher levels than those with the lowest amounts. Overall, among the 30 eggplant varieties, the macroelement content was higher in round eggplants than in long eggplants, while the trace element content was higher in long eggplants than in round eggplants. As few studies have explored the selection of mineral element content in eggplants, our study may provide additional foundations for superior eggplant breeding varieties.

An overview of all data collected confirmed a strong diversity in the appearance patterns of the 30 eggplant fruit samples evaluated. Karim et al. [42] reported that the total variation in the first three major axes of 10 morphological traits was 76.59% among 26 eggplant genotypes, which was broadly similar to the results of the present study. The appearance features that had the greatest effect on the total variation were peel color, peel brightness, and longitudinal diameter. This type of segregation among members related to fruit traits has also been described by other authors [43], which confirms that morphological variation of plant organs was widened during domestication [44]. Moreover, Alessandro et al. [45] reported 74% of total variance in 70 eggplants concerning genetic diversity. However, in our study, the diversity in the nutritional quality of the 30 eggplant varieties was weak, with the two principal components PC1 and PC2, explaining only 42.30% of the variance. The variables with the greatest influence on the total variance were organic acids, vitamin C, and hardness. In the study by Kumari et al. [46], the selected traits with the highest variation contribution on the first two axes of PCA (45.63% of variance) were total sugar, total chlorophyll, total phenolic content, and total antioxidant capacity. These differences may primarily result from diverse eggplant genotypes. Understanding the correlations between different traits is important for developing effective crop breeding programs, as a change in one trait can lead to changes in another trait [47]. Indeed, transverse diameter and hardness, and fruit weight and fruit shape index there were found to be positively correlated eggplant traits (*p* ≤ 0.01). This also indicates that among the 30 eggplant varieties, the round eggplants have a higher per fruit weight than long eggplants. Such associations were also reported by Datta et al. [48], who reported that the transverse diameter of eggplant fruits is positively correlated with mean fruit weight. Multiple sets of correlations were also found concerning the nutritional traits of the Chinese eggplant varieties. Malic acid being positively correlated with fructose and sucrose (*p* < 0.05), which can benefit the flavor quality of eggplant fruits, whereas oxalic acid showed a highly significant positive correlation with citric acid (*p* ≤ 0.01). Copper, zinc, K and magnesium were found to be positively correlated (*p* < 0.01), which indicated an additive effect between these indicators. Therefore, these traits can be used to create superior genotypes.

## 4. Materials and Methods

### 4.1. Plant Materials

The 30 eggplant varieties selected for this trial were introduced from 10 institutions across China. They were transplanted in September 2021 into a solar greenhouse at the Gobi Agricultural Park (Zongzhai Town, Suzhou District, Jiuquan City, China), and cultivated using a substrate method. Each commercial variety was planted with 20 seedlings, spaced at 45 cm between plants and 70 cm between rows. After flowering, fertilization was administered every 10 days via an irrigation system, with each application supplying 31.8 kg/ha of N, 11.55 kg/ha of P_2_O_5_, and 45.45 kg/ha of K_2_O. The cultivation area had an annual average temperature of 6.1 °C and received over 3000 h of sunlight. Samples were collected in November–December 2021 when the fruits reached commercial maturity. Twenty eggplant fruits of uniform size, similar maturity, and free of pests and diseases were selected from each variety for experimental analysis (Table 2).

### 4.2. Determination of Eggplant Fruit Appearance Characteristics

#### 4.2.1. Color and Fruit Calyx Thorns

According to the International Board for Plant Genetic Resources (IBPGR) 1990 [49], the agricultural morphological data of three traits of eggplant fruit, including fruit calyx thorn, pulp color, and peel color, have been scored and classified through quantitative and qualitative methods (Table S1). (Fruit calyx thorn: 0. no, 1. few, 2. moderate, and 3. many; Pulp color: 1. White, 3. Intermediate, 5. Green; Peel color: 1. Green, 2. Milk white, 3. Dark yellow, 4. Fire red, 5. Redish purple, 6. Greyish lilac, 7. Purple, 8. Blackpurple, 9. Black).

#### 4.2.2. Fruit Hardness

The hardness of four samples of each eggplant fruit variety was determined using a GY-4 Digital Fruit Hardness Tester (Zhejiang Toppan Yunnong Science and Technology Co., Shaoxing, China).

#### 4.2.3. Peel Brightness

The brightness of the peel of nine samples of eggplant fruits of each variety was measured using a CR-10 Plus colorimeter (Konica Minolta, Tokyo, Japan). The results are expressed as L (1–100, dark–black–bright–white) values.

#### 4.2.4. Longitudinal and Transverse Diameters, and Fruit Shape Index

The longitudinal and transverse diameters of six fruits from each eggplant variety were measured using a ruler. Fruit shape index was determined as the ratio between longitudinal and transverse diameters values.

### 4.3. Determination of Eggplant Fruit Quality

#### 4.3.1. Soluble Proteins, Vitamin C, and Nitrates Contents

The soluble protein content of the eggplant fruit was determined using the Coomassie brilliant blue method [50]. The 2,6-dichloroindophenol staining and salicylic acid methods were used to determine vitamin C and nitrate contents, respectively [51].

#### 4.3.2. Organic Acids Contents

Organic acid components were determined by high-performance liquid chromatography (HPLC) [52] using a Hi-PiexH (7.7 × 300 mm, 5 μm) chromatographic column and an ultraviolet detector. Briefly, the fresh eggplant samples were grinded and 5 g of homogenized samples were transferred to a 25 mL volumetric flask, rinsed with ultrapure water to a constant volume, vigorously shaken and mixed, and transferred to a 50 mL centrifuge tube. The samples were then centrifuged at 4 °C and 10,000 rpm for 10 min. The supernatant (2 mL) was filtered using a 0.22-μm microporous membrane and placed into a liquid chromatography sample injection bottle. The following analysis conditions were applied: 0.2 mmol·L^−1^ sodium dihydrogen phosphate as mobile phase, 0.5 mL·min^−1^ flow rate, and 20 μL of injection volume. The column temperature was set at 30 °C and the detection wavelength was 210 nm. Each eggplant fruit variety was analyzed thrice.

#### 4.3.3. Soluble Sugar Contents

The soluble sugar compounds in the eggplant fruits were determined by HPLC [52] using an LC-NH2 (250 × 4.6 mm, 5 μm) chromatographic column and a differential refractive detector. Briefly, fresh eggplant fruits were grinded into a homogenate, and 5 g were transferred into a 25 mL volumetric flask, rinsed with ultrapure water to a constant volume of 25 mL. All air within the samples was removed through sonication using a ultrasonic bath at 30 °C for 60 min. Then, the samples were centrifuged at 4 °C and 10,000 rpm for 10 min, and 2 mL of the supernatant was filtered using a 0.22-μm microporous membrane and placed into a liquid chromatography injection bottle. The following analysis conditions were applied: a acetonitrile:water (75:25) mixture was used as mobile phase, 1.5 mL·min^−1^ flow rate and 20 μL of injection volume. The chromatographic column temperature was set at 30 °C. Each eggplant fruit variety was analyzed thrice.

#### 4.3.4. Mineral Contents

Minerals in eggplant fruits were determined using a ZEEnit 700P atomic absorption spectrometer (Analytik Jena, Jena, Germany). Briefly, the eggplant fruits were fixed at 105 °C for 30 min and then dried at 80 °C until constant weight was achieved. The dried samples were then ground into powder and sieved through a 0.25 mm mesh. K, P, Ca, and Mg were extracted by digestion using the H_2_SO_4_/H_2_O_2_ wet digestion method. Cu, Fe, Mn, and Zn were extracted using the dry ashing method. Elemental P was determined using the molybdenum antimony colorimetric method [53].

### 4.4. Data Analysis

Data were analyzed by one-way analysis of variance using SPSS software (version 22.0; IBM Corp., Armonk, NY, USA) and Duncan’s multiple extreme variance test was used to compare statistically significant differences. Principal component analysis (PCA) and correlation analysis were performed using Origin 2021 software (OriginLab Corporation, Northampton, MA, USA). Results are expressed as mean ± standard error. In all analyses, probability values below 0.05 were considered statistically significant (*p* < 0.05).

## 5. Conclusions

In this study, the morphological characteristics and nutritional quality traits of 30 eggplant genotypes from China were evaluated. Descriptive statistical analysis revealed that the fruits varied the most regarding their fruit calyx thorn, peel brightness, and fruit shape index, whereas H’ index was found to be the largest concerning pulp color, followed by peel brightness, and the smallest concerning fruit weight. Significant differences in the nutritional qualities of the 30 Chinese eggplant fruits were observed. Moreover, the morphological characteristics of eggplants contributed the most to the diversity degree of the different samples, whereas the nutritional quality traits had a relatively small impact. In addition, there was a significant additive effect between various descriptive indicators of eggplant fruit that may be utilized for optimal selection. Hence, the herein identified donors of these traits may be utilized as parental genotypes to achieve genetic recombination and improve the morphological, agronomic, and fruit quality traits for eggplant breeding. Taken together, the findings of this study may pave the way for improved breeding strategies to achieve higher market value while maintaining high- quality standards for the final eggplant product.

## Figures and Tables

**Table 1:** Descriptive analysis of the fruit appearance characteristics of 30 Chinese eggplant varieties.

Index	Mean	SD	SE	Min.	Max.	CV (%)	H′ (%)
Fruit calyx thorn	1.60	1.07	0.20	0.00	3.00	66.87	1.30
Pulp color	2.33	0.76	0.14	1.00	4.00	32.49	1.45
Peel color	4.77	1.48	0.27	2.00	6.00	31.01	1.33
Peel brightness	25.04	15.36	2.81	14.18	62.01	61.36	1.41
Transverse diameter (cm)	4.46	1.64	0.30	2.37	8.57	36.68	1.34
Longitudinal diameter (cm)	14.72	3.44	0.63	6.88	22.23	23.37	1.25
Fruit shape index (%)	0.37	0.29	0.05	0.13	1.19	79.35	1.33
Per fruit weight (g)	309.40	90.16	16.46	173.30	476.50	29.14	1.16

SD, standard deviation; SE, standard error; CV, coefficient of variation; H′, Shannon–Weaver diversity index.

**Table 2:** Description of the 30 eggplant varieties investigated.

ID	Species	Form	Peel Color	Per Fruit Weight	Breeder Information
P1	1871	Round	Purple	210–230 g	Zhumadian Academy of Agricultural Sciences
P2	Zhengqie 924	Round	Purple	430–480 g	Zhengzhou Vegetable Research Institute
P3	420	Round	Purple	390–440 g	Institute of Vegetable and Flower Research, Chinese Academy of Agricultural Sciences
P4	cw213	Round	Purple	370–400 g	Institute of Vegetable and Flower Research, Chinese Academy of Agricultural Sciences
P5	1777	Round	Purple	375–405 g	Zhumadian Academy of Agricultural Sciences
P6	Zhengqie 907	Round	Green	450–490 g	Zhengzhou Vegetable Research Institute
P7	1942	Round	Green	410–460 g	Zhumadian Academy of Agricultural Sciences
P8	1908	Round	Green	420–465 g	Zhumadian Academy of Agricultural Sciences
P9	Zhengqie 908	Round	Green	410–450 g	Zhengzhou Vegetable Research Institute
P10	1919	Round	Green	400–435 g	Zhumadian Academy of Agricultural Sciences
P11	216	Long	Purple	320–370 g	Institute of Vegetable and Flower Research, Chinese Academy of Agricultural Sciences
P12	Yuzaoqie No. 9	Long	Purple	330–390 g	Chongqing Academy of Agricultural Sciences
P13	E160516	Long	Purple	150–205 g	Jilin Scientific Research Institute of Vegetables and Flowers
P14	19-824	Long	Purple	260–310 g	Chongqing Academy of Agricultural Sciences
P15	Weichangqie 101	Long	Purple	250–310 g	Weifang Academy of Agricultural Sciences, Shandong Province
P16	Yuqie No. 6	Long	Purple	155–200 g	Chongqing Academy of Agricultural Sciences
P17	Ron 19-14	Long	Purple	150–200 g	Chengdu Academy of Agriculture and Forestry
P18	Huhei 6	Long	Purple	200–260 g	Shanghai Academy of Agricultural Sciences
P19	Changqie 719	Long	Purple	230–280 g	Shandong Vegetable Institute
P20	Weichangqie 71	Long	Purple	320–380 g	Weifang Academy of Agricultural Sciences, Shandong Province
P21	Ron17-66	Long	Purple	150–200 g	Chengdu Academy of Agriculture and Forestry
P22	Weichangqie 78	Long	Purple	270–320 g	Weifang Academy of Agricultural Sciences, Shandong Province
P23	Huqie 316	Long	Purple	150–200 g	Shanghai Academy of Agricultural Sciences
P24	Changqie1016	Long	Purple	300–340 g	Shandong Vegetable Institute
P25	Zhengqie 809	Long	Purple	245–305 g	Zhengzhou Vegetable Research Institute
P26	E150725	Long	Purple	310–360 g	Jilin Scientific Research Institute of Vegetables and Flowers
P27	Zhengqie 903	Long	Purple	280–330 g	Zhengzhou Vegetable Research Institute
P28	1952	Long	Purple	280–320 g	Zhumadian Academy of Agricultural Sciences
P29	Lvtianshi	Long	Green	200–250 g	Shanghai Academy of Agricultural Sciences
P30	Wanqie 048	Long	Green	150–190 g	Anhui Academy of Agricultural Sciences, Institute of Horticulture

## Data Availability

Data are available from the corresponding author.

## Supplementary Materials

The following supporting information can be downloaded at: https://www.mdpi.com/article/10.3390/plants14121838/s1, Table S1: Morphological indicators of 30 eggplant varieties. Table S2: The sugar content of 30 eggplant varieties. Table S3: The content of organic acid components in 30 eggplant varieties.
