# Supplementary material for: Agronomic and Quality Traits of 30 Eggplant Germplasm Resources from China
Source: Plants (Basel). 2025 Jun 15;14(12):1838. doi: 10.3390/plants14121838 (PMC12196745; doi:10.3390/plants14121838)
Supplement: Supplementary file 1 [file plants-14-01838-s001.zip › plants-3581466-SI.pdf]

Table S1 Morphological indicators of 30 eggplant varieties

**Varieties**	**Fruit calyx thorn**	**Color of pulp**	**Color of peel**	**Pericarp brightness**	**Transverse diameter （cm）**	**Longitudinal diameter （cm）**	**Fruit shape index（%）**	**Fruit weight per fruit（g）**	**Hardness （kg·cm^{-2}）**
**P1**	0	3	8	15.7±1.01fgh	4.70±0.49ef	15.03±0.56efghij	0.31±0.02g	222.01±4.30jklm	5.17±0.43defgh
**P2**	1	3	5	21.01±1.63e	5.45±0.48cd	13.92±0.47ghijk	0.39±0.02def	353.64±20.43abc	5.11±0.29defghi
**P3**	3	1	5	18.26±0.57ef	8.15±0.67a	6.88±0.28n	1.19±0.04a	241.89±21.51bcde	4.97±0.36defghij
**P4**	2	3	5	18.27±0.86ef	8.55±0.63a	7.53±0.45n	1.14±0.04ab	368.51±22.98bcd	8.90±0.91a
**P5**	3	3	8	17.99±0.98f	8.57±0.68a	7.63±0.36n	1.13±0.04b	476.50±13.90bcde	6.77±1.10bcd
**P6**	3	5	2	49.36±1.88c	5.05±0.42de	13.58±0.66hijk	0.38±0.02ef	204.67±27.79a	6.50±0.55bcde
**P7**	3	1	1	47.56±2.19c	5.92±0.51bc	9.92±0.30m	0.60±0.02c	280.79±13.71abc	7.19±0.55bc
**P8**	3	3	2	53.49±0.87b	5.53±0.14cd	12.65±0.37kl	0.44±0.01def	273.06±19.45ab	5.15±0.42defghi
**P9**	3	5	2	54.44±0.77b	5.32±0.46d	13.27±1.02ijk	0.41±0.04de	185.76±17.10abc	6.68±0.76bcd
**P10**	2	1	3	42.88±1.13d	6.28±0.56b	11.10±0.55lm	0.57±0.04c	173.30±9.16abc	8.02±0.97ab
**P11**	1	3	8	16.22±0.88fgh	3.32±0.13jk	15.37±0.39efghij	0.22±0.00ij	238.98±12.47cdefg	3.83±0.07hijkl
**P12**	2	3	8	17.23±0.42fgh	3.33±0.25jk	16.70±0.82cdef	0.2±0.01ij	253.20±7.77bcdef	4.41±0.67fghijkl
**P13**	2	3	8	14.62±1.15gh	3.33±0.4jk	16.23±0.86cdefg	0.21±0.01ij	180.43±8.81m	3.14±0.17jkl
**P14**	2	3	8	17.01±0.92fgh	3.23±0.26jk	18.43±0.86bc	0.18±0.01jk	416.91±34.97ijklm	3.06±0.46kl
**P15**	0	3	8	15.69±1.07fgh	3.52±0.29ijk	17.12±0.55bcdef	0.21±0.01ij	422.85±15.04hijk	5.51±0.17cdefgh
**P16**	1	3	5	17.06±1.09fgh	3.95±0.31hi	17.82±1.03bcd	0.23±0.02ij	354.82±28.09hijkl	3.01±0.11kl
**P17**	2	3	8	17.27±0.47fgh	3.93±0.49hi	15.88±1.01defgh	0.25±0.01hi	183.16±18.93lm	4.12±0.23ghijkl
**P18**	0	3	5	14.18±1.07h	3.58±0.15ij	17.30±0.80bcde	0.21±0.01ij	437.58±17.31m	5.18±0.21defgh
**P19**	3	1	5	15.78±1.15fgh	4.22±0.37fgh	14.83±1.26fghijk	0.29±0.02gh	307.55±31.85hijkl	4.79±0.67efghijk
**P20**	0	1	8	17.20±0.49fgh	3.45±0.14ijk	15.75±0.69defgh	0.22±0.01ij	427.60±9.92cdefg	5.85±0.19cdefg
**P21**	1	3	5	15.99±1.56fgh	3.55±0.57ij	14.80±0.65fghijk	0.24±0.02hij	183.19±17.36lm	4.02±0.61ghijkl
**P22**	0	1	5	15.70±0.76fgh	3.47±0.22ijk	15.05±0.53efghij	0.23±0.01hij	378.08±34.49efghi	4.72±0.08efghijk
**P23**	2	5	5	14.31±1.33h	2.37±0.27l	19.03±1.55b	0.13±0.01k	310.14±5.08lm	3.29±0.12ijkl
**P24**	2	1	5	18.26±1.15ef	4.00±0.63ghi	16.50±0.67cdef	0.24±0.02hi	420.29±7.16efghi	3.22±0.30jkl
**P25**	2	1	8	17.66±0.69fg	3.63±0.31ij	14.9±0.75fghijk	0.25±0.02hi	290.57±25.3fghij	6.16±0.40cdef
**P26**	1	3	8	17.37±0.35fgh	3.60±0.35ij	17.37±0.79bcde	0.21±0.01ij	330.80±8.93defgh	2.75±0.26l
**P27**	1	3	5	17.77±0.61fg	4.48±0.41fg	13.08±0.44jkl	0.35±0.02fg	299.86±3.65fghij	5.37±0.33cdefgh
**P28**	2	3	8	17.54±0.71fg	3.27±0.44jk	15.6±0.76defghi	0.21±0.01ij	295.02±3.74fghij	4.34±0.48fghijkl
**P29**	0	3	2	53.37±1.79b	3.17±0.10jk	15.93±0.71defgh	0.20±0.01ij	390.31±4.35ijklm	7.09±0.46bc
**P30**	1	1	2	62.01±3.48a	2.97±0.19k	22.23±1.33a	0.13±0.01k	380.56±9.26lm	5.22±0.28defgh

According to the International Board for Plant Genetic Resources (IBPGR) 1990, the agricultural morphological data of three traits of eggplant fruit, including fruit ca-lyx thorn, pulp color, and peel color, have been scored and classified through quantita-tive and qualitative methods. (Fruit calyx thorn: 0.no, 1.few, 2.moderate, and 3.many; Pulp color: 1.White, 3.Intermediate, 5.Green; Peel color: 1.Green, 2.Milk white, 3.Dark yellow, 4.Fire red, 5.Redish purple, 6.Greyish lilac, 7.Purple, 8.Blackpurple, 9.Black).

Table S2 The sugar content of 30 eggplant varieties.

Varieties	Contents (mg·g^{-1} FW)		
	Fructose	Glucose	Sucrose
**P1**	13.64±0.09 e	12.93±0.06 c	13.6±0.09 a
**P2**	13.55±0.09e	8.53±0.09n	6.88±0.1g
**P3**	13.54±0.08e	10.34±0.08i	5.1±0.08j
**P4**	15.38±0.1 c	8.24±0.05o	11.66±0.09c
**P5**	13.15±0.1f	7.17±0.08q	5.81±0.08i
**P6**	17.12±0.07 a	9.5±0.08l	7.73±0.08f
**P7**	14.01±0.08d	10.17±0.09ij	3.98±0.08no
**P8**	11.81±0.08i	9.19±0.09m	5.29±0.03j
**P9**	12.95±0.09fg	8.12±0.06op	5.91±0.07i
**P10**	10.31±0.09kl	6.44±0.09r	5.92±0.08i
**P11**	12.69±0.07 g	15.19±0.09a	7.47±0.07f
**P12**	12.86±0.06 fg	9.92±0.09jk	5.93±0.09i
**P13**	10.14±0.08lm	11.18±0.08fg	12.03±0.07b
**P14**	13.16±0.06 f	10.82±0.05h	3.72±0.07o
**P15**	11.67±0.09i	9.78±0.07k	5.15±0.09 j
**P16**	12.89±0.07fg	13.65±0.08b	4.79±0.09k
**P17**	11.89±0.08 i	10.99±0.09gh	4.39±0.05lm
**P18**	12.26±0.1 h	11.53±0.02e	2.96±0.04p
**P19**	11.31±0.1 j	13.42±0.06b	5.65±0.09 i
**P20**	13.6±0.1e	11.34±0.09ef	5.2±0.1j
**P21**	10.53±0.07kl	9.89±0.08jk	10±0.03d
**P22**	7.02±0.09n	11.16±0.05fg	4.5±0.08l
**P23**	10.05±0.07 lm	13.58±0.08b	6.46±0.04h
**P24**	11.35±0.1j	7.88±0.07p	6.91±0.08g
**P25**	11.1±0.1 j	9.12±0.09m	6.38±0.09h
**P26**	13.79±0.1de	8.76±0.09n	4.12±0.08mn
**P27**	10.23±0.08kl	10.05±0.03ijk	2.57±0.06q
**P28**	9.88±0.09 m	11.28±0.09efg	9.72±0.06d
**P29**	16.41±0.09 b	11.95±0.09d	9.8±0.09d
**P30**	16.93±0.09a	11.25±0.06efg	8.14±0.07e

Table S3 The content of organic acid components in 30 eggplant varieties.

Varieties	Contents (mg·g^{-1} FW)				
	Oxalic acid	Malic acid	Quinic acid	Tartaric acid	Citric acid
**P1**	23.59±0.12ef	39.96±0.37gh	17.52±0.08c	0.96±0.01cde	0.61±0.01d
**P2**	10.79±0.14no	30.86±0.38n	6.49±0.05r	1.01±0.03cde	0.86±0.02b
**P3**	9.33±0.36o	58.45±0.76c	17.95±0.09b	1±0.09cde	0.57±0.02de
**P4**	26.52±0.34d	25.98±0.85o	17.95±0.04b	0.72±0.07fg	0.3±0.01jk
**P5**	15.92±0.22ijk	41.75±0.46fg	15.77±0.08g	0.99±0.02cde	0.36±0.03hij
**P6**	10.55±0.13no	38.42±0.85hij	22.04±0.04a	0.9±0.03de	0.43±0gh
**P7**	9.29±0.22o	34.74±0.54klm	15.35±0.09h	1.07±0.08cde	0.28±0.01kl
**P8**	17.66±0.14hi	51.73±0.64d	11.03±0.05n	0.46±0.02ij	0.27±0.01kl
**P9**	23.67±0.16ef	58.18±0.82c	16.51±0.07e	0.55±0.02ghij	0.4±0ghi
**P10**	23.65±0.49ef	23.16±0.94pq	4.38±0.08u	1.12±0.02c	0.51±0.01ef
**P11**	17.75±0.29hi	35.57±0.52kl	14.43±0.09j	0.73±0.02fg	0.32±0.03ijk
**P12**	16.91±0.18hij	42.9±0.43ef	16.22±0.04f	0.91±0.02de	0.28±0.01kl
**P13**	25.4±0.34de	62.28±0.98b	16.94±0.08d	0.7±0.01g	0.51±0.02ef
**P14**	20.2±0.14g	49.89±0.65d	6.76±0.09q	0.59±0.01ghij	0.32±0.03jk
**P15**	29.92±0.93c	20.89±0.65q	5.28±0.07t	1.57±0.06a	0.82±0.02b
**P16**	22.48±0.41f	45.11±0.5ef	13.29±0.03k	0.88±0.01ef	0.37±0.02hij
**P17**	18.41±0.76h	35.8±0.87jkl	9.29±0.08o	0.68±0.06gh	0.45±0.03fg
**P18**	25.74±0.82d	60.05±0.71bc	4.06±0.09v	1.53±0.02a	0.42±0.01gh
**P19**	14.86±0.17kl	23.82±0.13op	8.84±0.05p	0.59±0ghij	0.63±0.01d
**P20**	40±0.43a	23.67±0.96op	6.01±0.06s	0.67±0gh	0.45±0.04fg
**P21**	22.41±0.38f	57.87±0.97c	14.87±0.09i	1.36±0.03b	0.72±0.03c
**P22**	16.31±0.45ijk	30.26±0.74n	8.72±0.05p	0.42±0.06j	0.25±0.02kl
**P23**	13.26±0.3lm	35.84±0.39jkl	11.3±0.09m	0.49±0.03hij	0.31±0.01jk
**P24**	22.34±0.77f	34.13±0.89lm	15.3±0.09h	0.92±0.08de	0.31±0.01jk
**P25**	15.65±0.73jk	50.65±0.48d	16.69±0.04de	1.35±0.07b	1.31±0.03a
**P26**	14.73±0.67kl	39.09±0.34ghi	14.66±0.05ij	0.7±0.08g	0.43±0.01gh
**P27**	22.42±0.57f	40.81±0.26fgh	6.09±0.07s	0.6±0.08ghij	0.14±0.01n
**P28**	37.46±0.5b	83.62±0.65a	11.95±0.07l	0.56±0.01ghij	0.21±0.01lm
**P29**	20.36±0.21g	37.03±0.54ijk	14.7±0.06i	1.09±0.02cd	0.37±0.03hij
**P30**	11.75±0.59mn	32.72±0.82mn	13.41±0.09k	0.63±0.03ghi	0.17±0.01mn
